# Do our risk preferences change when we make decisions for others? A meta-analysis of self-other differences in decisions involving risk

**DOI:** 10.1371/journal.pone.0216566

**Published:** 2019-05-08

**Authors:** Eleonore Batteux, Eamonn Ferguson, Richard J. Tunney

**Affiliations:** 1 University of Nottingham, Nottingham, United Kingdom; 2 Aston University, Birmingham, United Kingdom; Universidad Loyola Andalucia, SPAIN

## Abstract

**Background:**

Are we more risk-averse or risk-seeking when we make decisions on behalf of other people as opposed to ourselves? So far, findings have not been able to provide a clear and consistent answer.

**Method:**

We propose a meta-analysis to assess whether self-other differences vary according to particular features of the decision. We reviewed 78 effect sizes from 49 studies (7,576 participants).

**Results:**

There was no overall self-other difference, but there were moderating effects of *domain* and *frame*. Decisions in the *interpersonal* domain were more risk-averse for self than for other. Decisions in the *medical* domain were more risk-seeking for self than for other. There were no overall self-other differences in the *financial* domain, however there was a moderating effect of *frame*: decisions in a *gain* frame were more risk-averse for self than other whereas decisions in a *loss* frame were more risk-seeking for self than other. This effect of *frame* was slightly different overall and in the medical domain, where self-other differences occurred in a *loss* frame but not in a *gain* frame.

**Conclusion:**

Future work should continue to investigate how the specific content and context of the decision impacts self-other differences in order to understand the effects of *domain* and *frame* we report.

## Introduction

### Background

The question addressed by the present meta-analytic review is the following: are we more risk-averse or risk-seeking for others compared to the self and is this context-dependent? The prospect of a risk is integral to the decisions we are faced with every day, meaning that investigating how people comprehend and react to the prospect of a risk is crucial to understanding the decisions they make. Although such research has largely focused on decisions that people make for themselves, there is a growing interest in investigating the decisions that people make on behalf of others (from individuals to societies)–surrogate decisions [1,2]. Indeed, we frequently make decisions for other people, such as buying a present for a loved one or preparing meals for our family. A range of professionals are also required to make risky surrogate decisions on a daily basis: doctors when selecting treatments for their patients for example. On a larger scale, financial investors and institutions often make risky decisions for other people which can have a role in global economic crises. The present meta-analysis provides an overview of the research to date, guided by Tunney and Ziegler’s [3] model of surrogate decision-making which allows us to bring some order to the literature and reframe it into a coherent, unifying account of self-other differences in risky decision-making.

#### Defining key terms

In keeping with the literature on self-other differences, we define a risky choice as having to decide under uncertainty, whereby at least one of the options contains a risky outcome. Risk can be expressed as a clear probability (e.g. 50% of chance of winning £100) or as an uncertain outcome (e.g. asking out a prospective partner). We do not conceptualise risk as necessarily denoting harm as other definitions might do [4]. For the purpose of this review, we establish surrogate decisions as involving on the one hand a decision-maker–the surrogate–and on the other a recipient–the person or people on behalf of which the surrogate is making a decision. We consider surrogate decisions as cases in which the recipient has no say in the decision process; it is not a negotiated decision. The recipient has no choice and accepts the outcome of the decision made by the surrogate. Decision-makers can vary in their relationship to the recipient, but in all cases, they make a decision for a recipient who is generally passive. There are cases of surrogate decision-making where the recipient and other parties are involved in negotiating the outcome, but this is a different type of decision and one we will not address here. We will also be confining our review to instances where the decision-maker actually makes a choice on behalf of the recipient, rather than giving advice or predicting their decisions.

Although the first few studies on self-other differences in risky decision making can be traced back to fifty years ago [5–7], there has been recent increased interest, particularly in the field of behavioural economics, linked to the involvement of such decisions in the financial crisis [8–10]. In the psychological literature, interest in surrogate decisions has grown against the backdrop of a long-standing interest in the role that emotions play in our decision-making [11–13]. In the medical field and particularly end-of-life care, the question has become of importance since reports that surrogate decision makers struggle to make accurate choices for their relatives [14]. However, results have often been contradictory, which may reflect the domain in which surrogate decisions are made and their impact on the decision-maker and recipient, amongst other factors. This means that there is no straightforward answer regarding whether we take more risk or less risk when we make decisions on behalf of other people as opposed to ourselves. The aim of this meta-analysis is to identify potential factors which contribute to the discrepancies in findings. Although a meta-analysis of self-other differences has been previously conducted [15], it only included studies prior to 2012 and is therefore missing a significant proportion of the literature. We wish to build on this review by using Tunney and Ziegler’s [3] model as a framework to guide our analysis.

### Theories and models of surrogate decision-making

Tunney and Ziegler’s [3] model of surrogate decision-making suggests a number of factors which may alter or bias the decision process that are of interest here. The identity of the recipient–who the decision is being made for–is expected to have an impact on the decision process. In effect, surrogate choices have been found to vary systematically from choices made for the self as psychological distance between the decision-maker and the recipient or outcome increases [16–18]. The significance or importance of the decision is also likely to play a role in that more thought and care would be put into more consequential decisions. Similarly, whether the decision-maker is held accountable or not is expected to increase the care put into a decision. Indeed, Pollmann, Potters and Trautmann [19] found a self-other difference in an investment task–surrogate decisions were more risk-taking–that disappeared with an accountability manipulation.

From a psychological perspective, the risk-as-feelings hypothesis suggests that our risk preferences are the product of an emotional reaction to the anticipated risk rather than a purely cognitive evaluation of the risk [12]. In a surrogate context, where the decision-maker is not the recipient of the outcome, there is an empathy gap between the decision-maker and the outcome [20], which might lead the decision-maker to underestimate the extent to which emotions affect others. This gap also creates psychological distance [21] between the decision-maker and the outcome, which means that they are more likely to engage in abstract rather than concrete thought. Therefore, one might expect that emotional involvement should be reduced in surrogate decision-making. In light of this, a straightforward prediction is that people’s own risk preferences will be *attenuated* when making a decision on behalf of someone else. We expect that surrogate risk preferences will be closer to risk-neutrality, which is consistent with research suggesting that surrogate decisions are more optimal than people’s own decisions–less susceptible to delay discounting [18], loss aversion [22] and framing effects [13]. However, the role that emotions play in the decision process is likely to vary according to features of the decision. For instance, emotional involvement may be stronger if the recipient is a child or a sibling rather than a stranger, or in a medical situation where the recipient’s life is at risk, it might be different than in financial situations; which is why examining how context affects surrogate decisions is important. Although the risk-as-feelings hypothesis supports the Tunney and Ziegler [3] model’s prediction about the identity of the recipient, it is unlikely to fully account for the occurrence and direction of self-other differences in all contexts.

Social Values Theory [23] proposes that surrogate decisions are made according to social values and expected appropriate behaviour. This arises from findings suggesting that people’s own choices take into account multiple factors whereas giving advice to others involves focusing on the most important factor of a decision [24]. Consequently, self-other differences will arise when there is a social value placed on taking or avoiding a risk. If taking a risk is socially valued, people will take more risk when making a decision for someone else than for themselves, and vice versa when risk-taking is not socially valued. It makes sense for surrogates to make choices according to social norms and values, particularly in cases where they are not familiar enough with the recipient to know what decision they would want to make. Social values add another layer of detail and complexity to the factors that influence self-other differences that is not necessarily accounted for by the Tunney and Ziegler [3] model. However, it is difficult to make predictions regarding the impact of social values on particular surrogate choices given that their existence and content is difficult to identify.

Taken together, the theories we have presented make numerous conjectures about the factors that influence surrogate decisions, thereby predicting that self-other differences present themselves differently under different circumstances. The benefit of conducting the present meta-analysis is to test whether these conjectures are supported. Indeed, findings regarding self-other differences in decisions involving risk have not always been consistent, which reinforces the need for a meta-analysis which investigates how different factors affect self-other differences.

### Findings on self-other differences in risky decision making

Self-other differences appear notably different between decision domains. In the interpersonal domain, decision-makers seem to be less risk averse when making hypothetical decisions for a friend than for themselves [23,25–27]. In the medical domain, physicians seem to be more risk averse when making hypothetical decisions for a patient as opposed to themselves, as do parents when making hypothetical decisions for their children [28–31]. However, in the financial domain, the literature is rather contradictory. There are findings suggesting that decision-makers are less risk averse for close and distant recipients [13,19,32–36], while others reporting that decision-makers are more risk averse for recipients [37,38], as well as findings reporting no self-other differences [39,40]. The aim of the meta-analysis will be two-fold: firstly, identifying whether self-other differences vary across domains and why that may be the case, and secondly, examining whether certain factors can explain the discrepancies in the financial domain.

### Moderators of self-other differences

We will first conduct a main analysis and moderator analyses of all effect sizes. We do not expect there to be an overall main self-other difference given that previous findings show that self-other differences in the medical and interpersonal domains are in opposite directions (therefore cancelling each other out when looking at an overall self-other difference) and results in the financial domain are mixed. Given our prediction that self-other differences in risk-taking vary across domains, we will conduct individual analyses for each decision domain to assess whether the context and content of the decision affects surrogate decisions differently in each domain. In order to do so, we also need to pick out theoretical moderators which we expect will have an influence on self-other differences given the theories we outlined above. Finally, to tease apart inconsistencies in findings, we will also include methodological moderators which can give us an indication of whether conflicting results are a consequence of experimental designs.

### Theoretical moderators

#### Domain

Given previous findings, we expect surrogates to take more risk for others than for themselves in the interpersonal domain, whereas we expect surrogates to take less risk for others in the medical domain. In the financial domain, we do not anticipate an overall self-other difference due to framing effects which we detail below. Furthermore, decisions in the medical and interpersonal domain can be more significant and life-changing than financial decisions that involve small amounts of money, which the literature overwhelmingly consists of. Social values and expectations may also be more prevalent in those domains. According to Tunney and Ziegler’s [3] model, self-other differences are indeed expected to vary across domains given that the significance of the decision, the accountability held upon the decision-maker and the intention of the decision-maker may vary. Social Values Theory [23] also predicts that self-other differences will differ across domains given that risk-taking is valued differently in each domain. Finally, we know that individual risk preferences are not constant across domains [41], nor do people attend to probabilities in the same way [42] or perceive the ratio between gains and losses to be equivalent [41]. It is therefore likely that self-other differences also vary across domains.

#### Frame

We expect self-other differences to differ depending on whether decisions are made in a gain or a loss frame. People tend to be risk averse in a gain frame and risk seeking in a loss frame [43]. According to the risk-as-feelings hypothesis which expects risk preferences to be attenuated when making decisions for others, we would expect self-other differences to be in opposite directions in a gain and a loss frame. We therefore predict that people take more risk for others in a gain frame and less risk for others in a loss frame, as has been found in previous studies [13,44,45]. In cases where decisions are framed as a gain but include the possibility of a loss, we speculate that self-other differences will be dampened compared to the gain frame. Similarly, for decisions that are framed as a loss but include the possibility of a gain, we expect that self-other differences will be dampened compared to the loss frame.

#### Recipient

Following from Tunney and Ziegler’s model [3], the impact of psychological distance [21] and empathy gaps [20], we expect the identity of the recipient of the surrogate decision to influence the decision process, thereby having an effect on self-other differences. Given past research mentioned above concerning the effect of psychological distance on surrogate decision-making, we predict self-other differences to be more pronounced when the recipient is a stranger than when the recipient is a close other (i.e. where a relationship has developed between the decision-maker and the recipient: friend, relative, long-term patient…). We speculate that self-other differences may disappear when decisions are made for a group because people might feel more accountable as the decision affects more people.

#### Accountability

We expect the level of accountability held against the decision-maker to have an effect on surrogate decisions, thereby making them more cautious and potentially reducing the risk that surrogates are willing to take. Indeed, it has been found that doctors make more conservative decisions for their patients than themselves due to fear of the legal consequences [28]. However, due to the low number of available studies that manipulated accountability, we did not use accountability as a moderator. We will instead draw tentative conclusions about its effect through an analysis of previous studies in our discussion.

### Methodological moderators

#### Decision outcome

In the financial domain, studies use either real decisions (performance-contingent payoffs) or hypothetical decisions (where the choices made in the experiment have no bearing on participant payment), which is why it is important to understand whether they are comparable. Data on whether the use of real or hypothetical rewards influences risk-taking is equivocal, with some studies reporting no difference [46,47], others reporting reduced risk-taking [48] or increased risk-taking [49] with real rewards. However, this has not been studied with respect to surrogate decision-making and the question remains open. Real decisions are likely to elicit stronger emotional involvement in the decision process than hypothetical decisions, or at least should better reflect a genuinely experienced emotion. Given that we assume self-other differences in risk preferences to be partly due to reduced emotional involvement when making a surrogate decision, we expect self-other differences to be larger when the outcomes are real rather than hypothetical. In terms of psychological distance, we know that there is greater distance between ‘near and far’ than ‘far and further’ [50]. If we consider hypothetical and surrogate decisions to be psychological distant decisions, surrogate decisions should be construed as more distant in real decisions than hypothetical decisions. We would therefore expect to find greater self-other differences in real than hypothetical decisions.

#### Design

We added the design used to measure self-other differences–whether the effect of recipient was elicited between-subjects or within-subjects–to investigate whether it moderates the strength of self-other differences. Within-subject designs might encourage participants to compare their decisions between recipients which could lead to experimenter demand effects whereby participants become aware of the experimental manipulation and change their behaviour, which could result in larger self-other differences (see [51] for a comparison of between- and within-subject designs in behavioural economics). On the other hand, within-subject designs could also lead to carry-over effects, in such a way that decisions in one condition could contaminate the other and therefore lead to a uniformisation of responses across conditions [52]. As mentioned by Charness *et al*. [51], carry-over effects do not tend to produce specific behavioural responses but are rather a function of the circumstances, whereas experimenter demand effects have a tendency to magnify differences between conditions. Given that demand effects make clearer predictions than carry-over effects, we expect within-subject designs to lead to stronger self-other differences than between-subject designs.

#### Publication status

We hypothesise that published studies will show larger self-other differences than unpublished studies as published studies are generally biased towards statistically significant results and present larger effect sizes as has been found in reviews comparing results from published and unpublished studies within meta-analyses [53,54].

## Method

### Search strategies

Various electronic databases were searched (Web of Science, PsycINFO, PubMed, Scopus, EconPapers, Science Direct, Social Science Research Network, Google Scholar, Google) to identify studies in March 2017 (we have also added relevant studies that have been published since). We used the following search terms: ‘risk’, ‘loss aversion’ or ‘uncertainty’; ‘self and other’, ‘self-other’, ‘other’, ‘surrogate’, ‘social distance’, or ‘psychological distance’; ‘choice’, ‘decision’ or ‘preference’. Studies that were cited by those that had been identified and studies that cited them were searched (backward and forward searching). Included studies compared choices (not ratings or advice) that an individual participant made for themselves to choices they made on behalf of another person or a group. Both published and unpublished studies (working papers, dissertations, doctoral theses, conference proceedings, unpublished data) were included. Unpublished studies were identified through the same search methods as published studies and we included some of our own unpublished data. Articles that were not written in English, French or Spanish were excluded.

**Fig 1:**
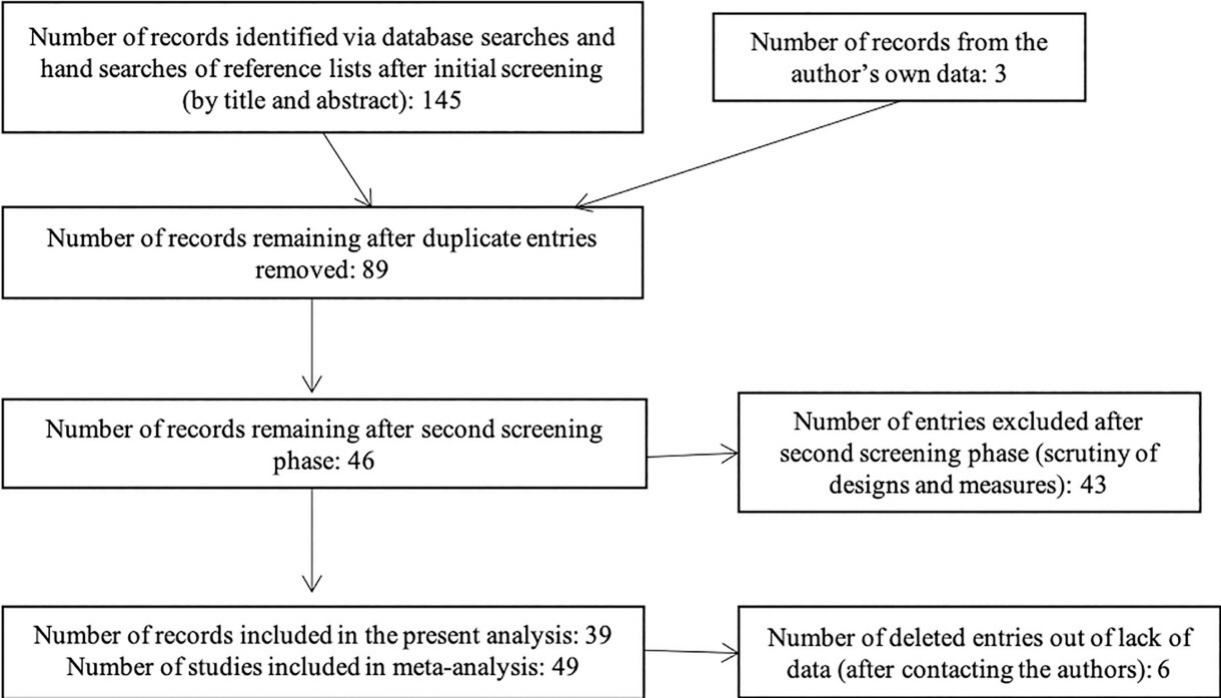
Flow of information through the different phases of the meta-analysis screening process, adapted from [55].

After screening records by title, abstracts of potentially relevant articles were examined (N = 145). Duplicates were removed (N = 59). The full text of the remaining articles was assessed for eligibility according to our criteria (N = 86) and articles that did not meet them were eliminated (N = 43). We ended up with 43 articles consisting of 49 studies, to which we added 6 unpublished studies (N = 55). We contacted authors of articles which did not include sufficient information to compute effect sizes and excluded 8 studies from 6 articles from authors who did not provide us with this information. We therefore included 49 studies with a total of 7576 participants and 72 effect sizes (see S1 for a list of studies and S1 for details of studies). Fig 1 contains details concerning the numbers of records identified through each screening phase, adapted from the PRISMA statement [55].

### Coding procedures

The first and third author read the papers independently and coded each study according to the coding frame developed by the first author. The percent agreement between both authors was high (89%). Disagreements were resolved through discussions between the first and third author. See S3 for details regarding the coding criteria used. Numbers associated to k refer to effect sizes.

#### Decision domain

We coded the domain in which participants were asked to make decisions: *Financial* domain (*k* = 54), *Interpersonal* domain (*k* = 9) or *Medical* domain (*k* = 15). We excluded studies in the interpersonal or medical domain that converted the outcomes of the choices that participants made to a monetary value which participants received as payment, as we believe this might have incentivised participants to think about their decisions as financial rather than medical/interpersonal, which makes the decision domain ambiguous.

#### Frame

Studies were coded according to whether decisions were made in a gain frame (k = 30), in a loss frame (k = 12), in a gain frame which included the possibility of a loss (k = 29) or in a loss frame which included the possibility of a gain (k = 7). In the financial domain, choices in a gain frame involved winning money, choices in a loss frame involved losing money, and choices which could either result in a loss or a win (investment tasks for example) were considered made in a gain frame with the possibility of a loss. In the medical domain, choices that involved taking a treatment to recover from an illness were coded as a loss with the possibility of a gain, as a gain could arise if the treatment works. Choices that involved a health improvement or vaccinations were coded as a gain or as a gain with the possibility of a loss, depending on whether doing so could worsen one’s health. In the interpersonal domain, choices which involved starting new relationships or moving relationships forward were coded as a gain with the possibility of a loss given the possible negative consequences of making such decisions (no studies included situations which could be coded as a loss).

#### Recipient

We coded whether the recipient of the surrogate decision was either a stranger or unidentified other (k = 39), a known/close other (friend, family member…) (k = 34), or a group of people (2 or more) (k = 5).

#### Decision outcome

We coded whether the outcome of the decision was hypothetical (k = 41) or real (k = 37). Real outcomes were studies where the recipient of the decision was affected by the decisions made (both the decision makers when making choices for themselves and the recipient when decision makers made surrogate choices). Studies which involved real outcomes when participants made decisions for themselves but hypothetical rewards when they made decisions for others were excluded.

#### Design

Studies were coded according to whether self-other differences were elicited using a between-subjects design (k = 34) or a within-subjects design (k = 44). In a between-subjects design, one group of participants made decisions for themselves, which was compared to another group of participants which made surrogate decisions. In a within-subjects design, the same group of participants made decisions for themselves as well as surrogate decisions.

#### Publication status

Studies were coded according to whether they were published studies (k = 51) or unpublished studies (k = 27).

### Computation of effect sizes

We used standardized mean differences (Cohen’s *d*) as the effect size metric. In a number of studies, effect sizes were estimated based on several assumptions. In cases where the total number of participants was given but not the exact number per group, we divided the total number by the number of groups to estimate the sample size. In cases where participants took part in two similar conditions (two different medical scenarios for example), we computed the effect size of both conditions together. When means and standard deviations were available, for between-subjects designs we used Cohen’s *d*_*s*_ and for within-subjects designs we used Cohen’s *d*_*av*_ [56]. For studies that only reported *t* values, for between-subjects designs we used Cohen’s *d*_*s*_ from *t* [57], and for within-subjects designs we used Cohen’s *d*_*z*_ [57]. For studies that only reported *F* values, we used Cohen’s *d*_*s*_ from *F* [58]. For studies that only reported *η*^*2*^, we transformed *η*^*2*^ to *d* [59]. For studies that reported the proportion of participants making a particular choice, we calculated the odds ratio which we converted to Cohen’s *d* [60]. We changed the sign of effect sizes where appropriate so that positive effect sizes represented choices for others that are more risk-taking than choices for the self and vice versa. We then transformed all effect sizes to Hedge’s *g* which corrects for biases in small samples and is recommended for use in meta-analyses [56].

### Analysis procedures

All analyses were performed in R using the *metaphor* package [61]. We used the random-effect model to compute the overall effect size of self-other differences rather than a fixed-effect model as the design and measures of included studies varied significantly. *I*^2^ and *Q* were used as measures of heterogeneity. We report the 95% confidence intervals of each effect size. The issue of publication bias was addressed via examining the funnel plot in which all effect sizes are plotted against the standard error. To evaluate the severity of potential publication bias we examined the effect size estimates following Duval and Tweedie’s [62] Trim-and-Fill method and Egger’s regression intercept [63]. We used mixed-effect models for the moderator analyses. To include a particular moderator or a sub-category of a moderator in an analysis, there had to be at least 3 effect sizes from independent studies in that category. Given that we expect to find self-other differences in different directions according to the decision domain, we conducted separate main and moderator analyses on each domain as well as an overall analysis. For studies that included multiple effect sizes that were not independent (different conditions in a within-subjects design for example), they only contributed one summary effect size for the main analysis. Summary effect sizes for these studies were computed using Cooper’s ‘shifting-unit-of-analysis’ method [64]. We did not use this method in moderator analyses as studies included multiple effect sizes because these related to different moderators; it did not make sense to compute a summary effect size in such cases.

## Results

### Analysis of all studies

**Fig 2:**
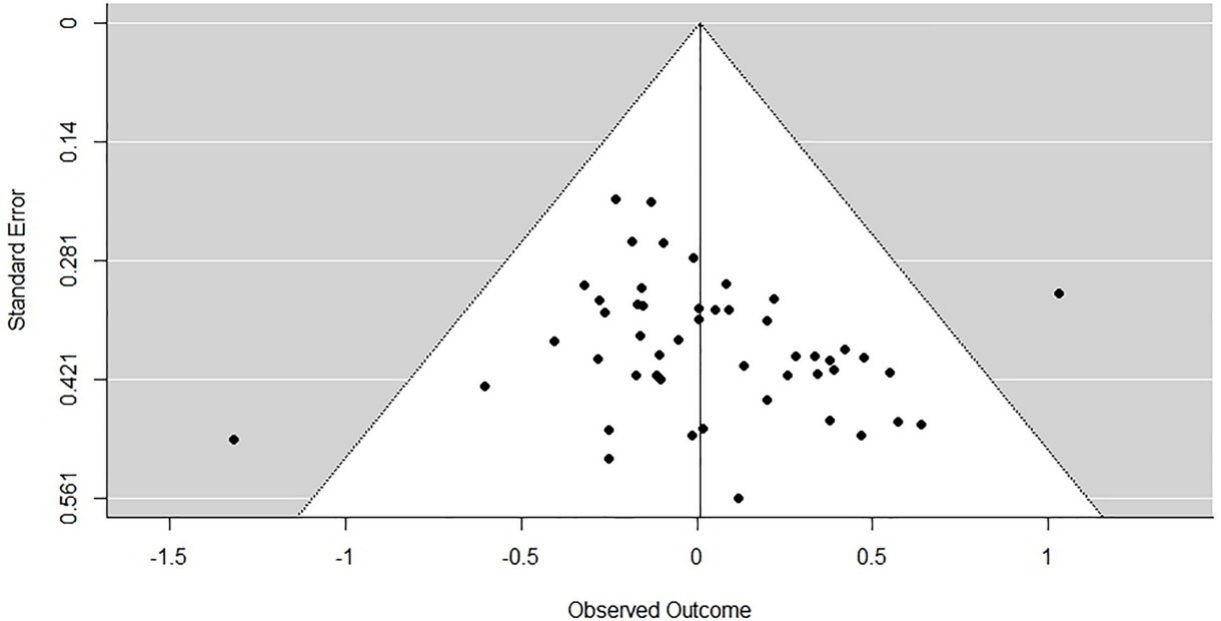
Funnel plot showing the effect sizes of all studies against their standard error. Effect sizes higher than 0 indicate that participants took more risk for someone else than for themselves. Effect sizes lower than 0 indicate that participants took less risk for someone else than for themselves.

Across all the studies there were no self-other differences in risk-taking (*k =* 49, *g* = 0.009, CI (-0.092, 0.109), *p* = .864). The analysis revealed that effect sizes were roughly symmetrical (Tau-squared = 0) and an absence of heterogeneity (*I*^2^ = 0, *Q* = 42.281, *p* = .705). We assessed the extent of publication bias by firstly examining the adjusted effect size estimates according to the Trim-and-Fill procedure with a random effects model. No studies were found missing above the average effect size estimate, but ten studies were found missing below the average effect size. When ten studies with an imputed effect size lower than the mean effect estimate were filled in, the effect size estimate was in the other direction (*k =* 59, *g* = -0.089, CI (-0.194, 0.158), *p* = .096). However, Egger’s regression test for funnel plot asymmetry revealed a non-significant regression coefficient (intercept = -0.13, SE = 0.12, *p* = .258). Taken together, both indicators suggest that publication bias has probably not affected the present analysis. See Fig 2 for the funnel plot.

#### Moderator analyses

The analysis revealed that the decision domain significantly moderated self-other differences in risk-taking (*Q* = 26.732, *p* < .001). There were no self-other differences in the financial domain (*k* = 54, *g* = 0.010, I (-0.099, 0.120), *p* = .852). However, there were significant differences in the interpersonal domain (*k* = 9, *g* = 0.554, CI (0.285, 0.823), *p* < .001) and in the medical domain (*k* = 15, *g* = -0.267, CI (-0.430, -0.105), *p* = .001), meaning that surrogate decisions were more risk-taking in the relationship domain but less risk-taking in the medical domain. The frame of the decision was also a significant moderator (*Q* = 13.531, *p* = .009). There were no self-other differences in the gain frame (*k* = 30, *g* = 0.063, CI (-0.078, 0.204), *p* = .379) and no self-other differences in the gain frame which included the possibility of a loss (*k* = 29, *g* = 0.101, CI (-0.040, 0.242), *p* = .159). There were differences in the loss frame (*k* = 12, *g* = -0.264, CI (-0.507, -0.022), *p* = .033) and in the loss frame which included the possibility of a loss (*k* = 7, *g* = -0.300, CI (-0.535, -0.064), *p* = .013) where decisions were less risk-taking for others than for the self. The remaining variables (recipient, outcome, design, publication status) were not significant moderators. A statistical breakdown of these results can be found in S4.

#### Meta-regression

We conducted a meta-regression with all the above moderators (domain, frame, recipient, outcome, design, publication) which was significant (k = 78, *Q* = 36.199, *p* < .001). Full results can be found in Table 1. The difference between the interpersonal and the financial domain was significant (*B* = 0.525, *p* = .014). The difference between the medical and the financial domain was marginally significant (*B* = -0.355, *p* = .057). The difference between the gain and loss frame was significant (*B* = -0.441, *p* = .004).

**Table 1:** Meta-regression on analysis of all studies*.

	B	SE	95% CI	P
Intercept	0.213	0.131	-0.044, 0.470	.105
Relationship (1) vs Financial (0)	0.525	0.214	0.106, 0.944	**.014**
Medical (1) vs Financial (0)	-0.355	0.186	-0.720, 0.010	.057
Loss (1) vs Gain (0)	-0.441	0.154	-0.743, -0.139	**.004**
Gain with Loss (1) vs Gain (0)	-0.161	0.118	-0.392, 0.071	.174
Loss with Gain (1) vs Gain (0)	-0.132	0.179	-0.482, 0.218	.459
Close (1) vs Stranger (0)	-0.064	0.116	-0.291, 0.162	.578
Group (1) vs Stranger (0)	-0.151	0.200	-0.543, 0.241	.450
Hypothetical (1) vs Real (0)	0.037	0.146	-0.250, 0.325	.798
Within (1) vs Between (0)	-0.031	0.100	-0.226, 0.165	.759
Unpublished (1) vs Published (0)	-0.030	0.107	-0.240, 0.180	.782

*Note: model is significant (p < .001)

### Interpersonal domain

Across all studies in the interpersonal domain there were significant self-other differences in risk-taking (*k =* 8, *g* = 0.571, CI (0.296, 0.847), *p* < .001), indicating that people take more risk for another person than for themselves. The analysis revealed that there was an absence of variation in the distribution of effect sizes (Tau-squared = 0) as well as an absence of heterogeneity (*I*^2^ = 0, *Q* = 3.838, *p* = .798). We assessed the extent of publication bias by firstly examining the adjusted effect size estimates according to the Trim-and-Fill procedure with a random effects model. No studies were found missing below the average effect size estimate but two studies were deemed missing above the average effect size estimate. When two studies with an imputed effect size greater than the mean effect estimate were filled in, the effect size estimate was slightly higher (*k =* 10, *g* = 0.657, CI (0.409, 0.906), *p* < .001). This suggests that the analysis may be biased towards understating the summary effect size. This was confirmed by using Egger’s regression test for funnel plot asymmetry which revealed a significant regression coefficient (intercept = 1.46, SE = 0.37, *p* = .008). Both indicators suggest that publication bias has affected the analysis by weakening the effect. We were unable to conduct moderator analyses on decisions from the interpersonal domain due to a low number of effect sizes.

### Medical domain

Across all studies in the medical domain there were significant self-other differences in risk-taking (*k =* 11, *g* = -0.297, CI (-0.481, -0.112), *p* = .002), indicating that people take less risk for others than for themselves. The analysis revealed that there was an absence of variation in the distribution of effect sizes (Tau-squared = 0) as well as an absence of heterogeneity (*I*^2^ = 0, *Q* = 7.231, *p* = .703). We assessed the extent of publication bias by firstly examining the adjusted effect size estimates according to Duval and Tweedie’s (2000) Trim-and-Fill procedure with a random effects model. No studies were found missing below the average effect size estimate, but four studies were found missing above the average effect size estimate. When four studies with an imputed effect size higher than the mean effect estimate were filled in, the effect size estimate was slightly lower (*k =* 15, *g* = -0.194, CI (-0.363, -0.026), *p* = .026). However, Egger’s regression test for funnel plot asymmetry did not reveal a significant regression coefficient (intercept = -0.02, SE = 0.12, *p* = .897). Therefore, we can conclude that the present analysis is probably not contaminated by publication bias.

We found that the frame of the decision was a significant moderator (*Q* = 8.391, *p* = .015). Self-other differences in a gain frame were not significant (k = 6, g = -0.203, CI (-0.474, 0.068), p = .141), but they were in a loss frame with the possibility of a gain (k = 7, g = -0.300, CI (-0.535, -0.064), p = .013) where risk-taking was higher for self than other. There were no decisions made in a loss frame and not enough made in a gain frame with the possibility of a loss to include it in the analysis. The recipient of the surrogate decision was a significant moderator (*Q* = 10.470, *p* = .005). Self-other differences when decisions were made on behalf of a stranger were not significant (k = 4, g = -0.319, CI (-0.776, 0.138), p = .171), but they were significant when decisions were made on behalf of a close other (k = 11, g = -0.260, CI (-0.434, -0.086), p = .003). There were self-other differences when decisions were made on behalf of a close other meaning that decisions were less risk-seeking for a close other than for a stranger (there were no studies where decisions were made on behalf of a group). Finally, the design was a significant moderator (*Q* = 10.657, *p* = .005), whereby self-other differences were larger in a within-subjects design (k = 10, g = -0.310, CI (-0.543, -0.076), p = .009) than a between-subjects design (k = 5, g = -0.228, CI (-0.454, -0.001), p = .049). We could not perform moderator analyses on outcome and publication status due to a low number of effect sizes. Given this, we did not perform a meta-regression either.

### Financial domain

Across all studies in the financial domain there were no significant self-other differences in risk-taking (*k =* 31, *g* = 0.036, CI (-0.095, 0.167), *p* = .594). The analysis revealed that there was an absence of variation in the distribution of effect sizes (Tau-squared = 0) as well as an absence of heterogeneity (*I*^2^ = 0, *Q* = 11.433, *p* = .999). We assessed the extent of publication bias by firstly examining the adjusted effect size estimates according to the Trim-and-Fill procedure with a random effects model. No studies were found missing above the average effect size, but four studies were found missing below. When four studies with an imputed effect size greater than the mean effect estimate were filled in, the effect size estimate was slightly lower (*k =* 35, *g* = -0.011, CI (-0.136, 0.115), *p* = .866). However, Egger’s regression test for funnel plot asymmetry did not reveal a significant regression coefficient (intercept = -0.20, SE = 0.11, *p* = .088). Therefore, we can conclude that the present analysis was probably not contaminated by publication bias.

The frame of the decision was a significant moderator (*Q* = 8.323, *p* = .040). Self-other differences in the gain frame were marginally significant (*k* = 24, *g* = 0.163, CI (-0.003, 0.328), *p* = .054) where people took slightly more risk for others than for themselves. There were self-other differences in the loss frame (*k* = 12, *g* = -0.264, CI (-0.507, -0.022), *p* = .033) where people took less risk for others than for themselves. However, there were no self-other differences in the gain frame when the choice included the possibility of a loss (*k* = 18, *g* = -0.020, CI (-0.202, 0.162), *p* = .831). The remaining variables (recipient, outcome, design, publication status) were not significant moderators. A statistical breakdown can be found in S4.

Finally, we conducted a meta-regression with all the above moderators (domain, frame, recipient, outcome, design, publication status) which approached significance (k = 54, *Q* = 10.179, *p* = .179). Results can be found in Table 2, in which it can be seen that, even though the model is not significant, there is a difference between decisions made in a gain frame as opposed to those made in a loss frame (*B* = -0.452, *p* = .004).

**Table 2:** Meta-regression on analysis of financial domain*.

	B	SE	95% CI	p
Intercept	0.276	0.137	-0.035, 0.503	.089
Loss (1) vs Gain (0)	-0.452	0.157	-0.760, -0.144	**.004**
Gain with Loss (1) vs Gain (0)	-0.176	0.133	-0.437, 0.086	.188
Close (1) vs Stranger (0)	-0.152	0.136	-0.419, 0.115	.264
Group (1) vs Stranger (0)	-0.165	0.201	-0.559, 0.228	.410
Hypothetical (1) vs Real (0)	0.075	0.150	-0.219, 0.369	.618
Within (1) vs Between (0)	-0.012	0.122	-0.250, 0.236	.920
Unpublished (1) vs Published (0)	-0.047	0.117	-0.276, 0.183	.689

*Note: model is not significant (p = .179)

## Discussion

We did not find an overall self-other difference. However, we show that distinct patterns of self-other differences emerge when we consider a series of theoretical moderators. Crucially, we found that there are differences between decision domains and decision frames, even when other moderators are accounted for. This suggests that self-other differences are not easily comparable and sheds light on the inconsistencies in findings that have arisen so far. In the sections below, we discuss these moderator effects.

### Domain

Self-other decisions were moderated by decision domain (medical, financial or interpersonal). In the medical domain, decision-makers are more risk-taking for themselves than for another person (small effect), whereas in the interpersonal domain they are more risk-taking for someone else than for themselves (medium effect). In the financial domain, there seems to be an overall absence of self-other differences. This finding is concurrent with the previous meta-analysis [15] which showed that decisions were more risk-averse for others in a medical context but that there were no self-other differences in other contexts (financial and interpersonal decisions were analysed together). This is crucial to our understanding of surrogate decision-making as it shows that decisions are not necessarily comparable across domains, meaning that there are features of each domain which require further investigation to understand why divergent patterns of self-other differences arise.

A key difference between the interpersonal and medical domains and the financial domain is the significance of the decision, which could explain the difference in effect size between domains. Although financial decisions can be just as important and consequential as interpersonal or medical decisions, the amounts of money that are used in all included studies but one (study 3 in [9] are small (two or three digit amounts). In fact, studies that use real rewards (about two thirds of financial studies) convert outcomes of choices to payments (which tend to be a single digit amount), meaning that participants are actually making decisions that involve very small amounts of money. All financial studies used relatively inconsequential decisions, whereas interpersonal studies could include life-changing decisions and medical studies often did. The present meta-analysis can draw conclusions only about financial decisions which have a small outcome. It may be that self-other differences are altered when large outcomes are studied. On the other hand, a significant proportion of studies in the financial domain used real decisions, thereby increasing their ecological validity. In the medical and interpersonal domain, where decisions were hypothetical, it could be that participants were motivated by self-image concerns, conforming to social norms in an experimental setting, but might not do so in real scenarios. However, we did not find any differences between real and hypothetical outcomes in financial decisions.

Following from Social Values Theory [23], it could be the case that risk-aversion is valued in the medical domain whereas risk-taking is valued in the interpersonal domain. It is plausible to assume that taking a medical decision that could lead to a negative outcome, or the absence of an outcome, would be seen as a bad decision. A high degree of responsibility and accountability could be then held against the decision-maker, particularly in public health scenarios or decisions that could lead to the death of a patient. In terms of relationships however, taking a certain level of risk is perhaps necessary to developing a relationship. It remains unclear which social values could prevail in financial decisions, particularly those with little consequences. Nonetheless, taking high risks could be considered impulsive and irrational, and therefore not socially valued, especially when it comes to decisions which involve large amounts of money. However, in the case of the financial decisions made leading up to the financial crisis, decision-makers were in fact accused of excessive risk-taking [8], which makes the study of surrogate financial decisions with large outcomes all the more relevant and necessary.

### Frame

We also found that the frame of the decision was a significant moderator, but did not necessarily manifest itself in the same way across domains. Overall, people were less risk-taking for someone else than for themselves in a loss frame and in a loss frame which includes the possibility of a gain. There were no differences when decisions were in a gain frame or in a gain frame which included the possibility of a loss. This is consistent with the previous meta-analysis [15] which found self-other differences for losses but not gains. Given that we know that people are more impacted by losses than gains [43], it is plausible that this effect would also translate to a surrogate context, where accountability might be higher for losses than gains. In the financial domain, self-other differences were divergent depending on the frame in which they were elicited and followed the predictions we made given the risk-as-feelings hypothesis (i.e. risk preferences were attenuated in a surrogate context). This helps to elucidate contradictory findings in the financial domain. In the medical domain, although risk-taking was reduced for others, self-other differences were significant in a loss frame but not in a gain frame. We were unable to investigate the effect of frame in the interpersonal domain due to a lack of studies made in a loss frame. In fact, all studies from the interpersonal domain used or adapted the scenarios devised by Beisswanger *et al*. [25], which could be why the most consistent and strongest self-other difference is found within this domain. There is therefore a need for studies using different scenarios to study the interpersonal domain.

### Recipient

We did not find that the identity of the recipient of the surrogate decision moderated the overall effect, although this could be because there was no overall self-other difference. In the medical domain, the recipient was a significant moderator of the self-other difference: people take less risk when decisions were made on behalf of a close other than on behalf of a stranger. This could be because they are more concerned about taking a risk which leads to a negative outcome when the recipient is someone close to them as opposed to someone they do not know. It could also be that accountability has a larger effect on decisions for a close other than for a stranger, if decisions made for a stranger give more anonymity to the decision-maker for example. In the interpersonal domain, we were unable to look at the effect of recipient. In the financial domain, although the identity of the surrogate recipient was not a significant moderator, the effect sizes indicate a trend whereby decisions made on behalf of a stranger may be more risk-taking than one’s own decisions, whereas decisions made for a close other or a group of people may be less risk-taking. We therefore find some evidence for an effect of psychological distance, but evidence is weak and further research is needed. Interestingly, a study found that surrogates believe they would reduce their financial risk-taking for others relative to themselves [65], which is in line with the behavioural trend we find here for close others but not distant others. This indicates that there might be a discrepancy between what surrogates believe they would do and what they actually do. There is also evidence from the wider literature that psychological distance has an effect on surrogate decisions. For example, smaller psychological distance increases surrogates’ emotional burden, making them more likely to minimise the risk of regret when making decisions [66].

### Accountability

We could not quantitatively assess the effect of accountability given the low number of studies that we were able to include. The results of these studies are nonetheless interesting. Eriksen and Kvaloy [37] found that people take less risk for someone else than for themselves in an investment task where the recipient was given feedback on choices made by the decision-maker. Pollmann *et al*. [19] found that people take less risk for others when accountability is manipulated in an investment task than when it is not. Losecaat Vermeer [67] found that in a gain frame, people took more risk for someone else but more so in a low than a high responsibility condition. Moreover, in terms of decision made on behalf of a group of people, whereby the decision is affecting more people and therefore has larger consequences, we hypothesise that this would increase the effect of accountability and social values on the decision process and perhaps also reduce risk-taking, which is in line with the trend evidence reported here. This is in line with the previous meta-analysis which suggests that surrogate decisions might be drawn to risk-aversion due to the avoidance of anticipated blame [15]. More studies are needed to investigate the effect of accountability on risk-taking in the financial literature, and it would be particularly interesting to assess its impact on decisions with large outcomes–perhaps holding decision makers accountable for their decisions can reduce irresponsible or high risk-taking. For similar reasons, assessing its impact on other decision domains would be beneficial, such as studying the consequences that the fear of being held accountable or legally pursued can have on doctors’ decision-making.

### Methodological moderators

We did not find that the nature of the outcome impacted self-other differences. This is particularly relevant to the financial domain where there is an ongoing debate about the validity of experiments that do not use performance-contingent payments [68]. Although risk-taking might differ between real and hypothetical rewards, this does not seem to affect the conclusions that can be drawn about self-other differences. However, we were not able to include real medical or interpersonal decisions. We did not find differences overall between studies that used a between-subject design to test self-other differences and those that used a within-subjects design. On the other hand, self-other differences in the medical domain were stronger in within-subject designs than in between-subject designs, which indicates some evidence for experimenter demand effects. We did not find differences either between published and unpublished studies, which is reassuring with respect to potential publication bias. However, this could be partly attributable to the fact that a lot of studies were working papers from the economics literature which are of a similar standard to published papers.

### Future directions

The present meta-analysis has identified several gaps in the literature. For a start, there are considerably more studies that have investigated financial decisions than interpersonal and medical decisions. We could also not perform several of our moderator analyses due to a low number of effect sizes. Further work is needed to identify how the identity of the surrogate recipient affects decisions in both the interpersonal and the financial domains. To be able to adequately compare self-other differences across domains, decisions in the financial domain that have higher levels of significance require investigation. One of the setbacks to studying larger outcomes is that using real payoffs that match amounts used in the experimental task is unlikely to be possible. However, we did not find a difference here between studies using real and hypothetical outcomes, indicating that perhaps using hypothetical outcomes may be an adequate proxy for real decisions. Nevertheless, real-world decisions are often a lot more complex than the scenarios set up in these studies, which is why it is so important to study how a variety of features of a decision impact surrogate risk-taking. There is indeed a need for looking at surrogate decisions in real world settings, particularly for medical and interpersonal domains where experimental studies have so far been restricted to hypothetical scenarios. Investigating the role of accountability and social values will be a particularly important step to understanding real-world decision-making.

Finally, in this review, we have been quite liberal about our definition of risk and the studies we incorporated as a result in our meta-analysis. We chose to keep the definition broad in order to bring together different literatures and theories on risk-taking in surrogate decision-making. These can be quite distinct in the scenarios they present. For example, experiments in the economic literature uses probabilistic outcomes whereas those in the interpersonal did not and conceptualised risk as the uncertainty contained in the actions of others. For that reason, we analysed these literatures together in our overall analysis, but also separately by decision domain. Nevertheless, the differences between the scenarios in each domain leads one to wonder whether they are measuring the same thing when it comes to risk. Future work should aim to create scenarios which are more easily comparable across domains, controlling for factors such as the presence or absence of clear probabilistic outcomes. There is indeed evidence of a distinction between our attitudes towards a purely probabilistic risk (where risk is not contingent on others’ behaviour) and a social risk (where risk is contingent on others’ behaviour) [69], which is reflected in the finding that individual risk attitudes vary between domains [41]. Future work should aim to tease apart both types of risk in the context of surrogate decisions in order to better understand the domain differences we highlight here.

### Conclusion

Our meta-analysis indicates that the differences between risky decisions that people make for themselves and those they make for others vary according to the domain and the frame of the decision. We believe that the present meta-analysis has contributed to the debate in the literature and offered potential avenues of research to be pursued to enhance our understanding of risk preferences in surrogate decision making.

## Supporting information

S1 Appendix List of studies. (DOCX)

S2 Appendix Characteristics and effect sizes of all studies. (DOCX)

S3 Appendix Coding frame for methodological and theoretical moderators. (DOCX)

S4 Appendix Statistical differences between self and other decisions. (DOCX)

S5 Appendix PRISMA checklist. (DOC)
