# Supplementary material for: Do our risk preferences change when we make decisions for others? A meta-analysis of self-other differences in decisions involving risk
Source: PLoS One. 2019 May 8;14(5):e0216566. doi: 10.1371/journal.pone.0216566 (PMC6505775; doi:10.1371/journal.pone.0216566)
Supplement: S1 Appendix — (DOCX) [file pone.0216566.s001.docx]

Supplementary File 1: List of papers included in meta-analysis

1. Andersson, O., Holm, H. J., Tyran, J.-R., & Wengström, E. (2013). Risking Other People’s Money: Experimental Evidence on Bonus Schemes, Competition, and Altruism. IFN Working Paper, (989), 1–49.
2. Andersson, O., Holm, H. J., Tyran, J.-R., & Wengström, E. (2014). Deciding for Others Reduces Loss Aversion. Management Science, 2461(13), 0–38. <https://doi.org/http://dx.doi.org/10.1287/mnsc.2014.2085>
3. Batteux, E., Ferguson, E., & Tunney, R. J. (2017a). Risk Preferences in Surrogate Decision Making. Experimental Psychology, 64, 290–297. <https://doi.org/10.1027/1618-3169/a000371>
4. Batteux, E., Ferguson, E., & Tunney, R. J. (2017b). Risk preferences in financial and medical surrogate decision making. Unpublished data.
5. Batteux, E., Ferguson, E., & Tunney, R. J. (2019). Do we make decisions for other people based on our predictions of their preferences? Evidence from financial and medical scenarios involving risk. Thinking & Reasoning. <http://doi.org/10.1080/13546783.2019.1592779>
6. Beisswanger, A. H., Stone, E. R., Hupp, J. M., & Allgaier, L. (2003). Risk Taking in Relationships: Differences in Deciding for Oneself Versus for a Friend. Basic and Applied Social Psychology, 25(2), 137–143. <https://doi.org/10.1207/S15324834BASP2502>
7. Benjamin, A. M., & Robbins, S. J. (2007). The role of framing effects in performance on the Balloon Analogue Risk Task (BART). Personality and Individual Differences, 43(2), 221–230. <https://doi.org/10.1016/j.paid.2006.11.026>
8. Carroll, A. E., Saha, C., Ofner, S., & Downs, S. M. (2017). Valuing health for oneself versus one’s child or elderly parent. Journal of Health Psychology. <https://doi.org/10.1177/1359105317712574>
9. Carstensdottir, G. (2015). Bursting Your Balloon: Examining Differences in Self-Other Decision Making Using the BART Task. Available at ﻿<https://skemman.is/handle/1946/22499?locale=en>
10. Colby, H. A. (2010). Risk preferences in surrogate financial decision making. Available at ﻿<https://rucore.libraries.rutgers.edu/rutgers-lib/27268/>
11. ﻿Dore, R. A., Stone, E. R., & Buchanan, C. M. (2014). A Social Values Analysis of Parental Decision Making, The Journal of Psychology, 148:4, 477-504. <https://doi.org/10.1080/00223980.2013.808603>
12. Eriksen, K. W., & Kvaløy, O. (2010). Myopic investment management. Review of Finance, 14(3), 521–542. <https://doi.org/10.1093/rof/rfp019>
13. Eriksen, K. W., Kvaløy, O., & Luzuriaga, M. (2017). Risk-taking on behalf of others. Working Paper, (February). Retrieved from <https://ideas.repec.org/p/ces/ceswps/_6378.html>
14. Fleming, A., & Slank, K. (2015). Making a choice: self-other differences in decision making in risky situations. North American Journal of Psychology, 17(3), 633-648. ﻿<http://dx.doi.org/10.1108/17506200710779521>
15. Füllbrunn, S., & Luhan, W. J. (2015). Am I my peer’s keeper ? Social Responsibility in Financial Decision Making. Working Paper.
16. Garcia-Retamero, R., & Galesic, M. (2012). Doc, What Would You Do If You Were Me? On Self-Other Discrepancies in Medical Decision Making. Journal of Experimental Psychology-Applied, 18(1), 38–51. [https://doi.org/Doi 10.1037/A0026018](https://doi.org/Doi%2010.1037/A0026018)
17. Haavik, M., & Zeiler, S. (2010). Risk-taking on behalf of others: A laboratory experiment. Available at ﻿<https://brage.bibsys.no/xmlui/handle/11250/183783>
18. Humphrey, S., & Renner, E. (2011). The social costs of responsibility. Working Paper. Available at ﻿<https://ideas.repec.org/p/cdx/dpaper/2011-02.html>
19. Lu, J., Shang, X., Li, B. (2018). Self–Other Differences in Decision-Making Under Risk. Experimental Psychology, 65(4), 226-235. https://doi.org/10.1027/1618-3169/a000404
20. Montinari, N., & Rancan, M. (2013). Social preferences under risk: the role of social distance. Working Paper. Available at ﻿<http://cadmus.eui.eu/handle/1814/29220>
21. Oliver, A. (2013). Testing the rate of preference reversal in personal and social decision-making. Journal of Health Economics, 32(6), 1250-1257. ﻿<http://dx.doi.org/10.1016/j.jhealeco.2013.10.003>
22. Palmer, E. C. (2014). Self-other differeces in risky decision making: an analysis of social values theory and construal-level theory. Available at ﻿<https://wakespace.lib.wfu.edu/handle/10339/39283>
23. Petrova, D., Garcia-Retamero, R., Van Der Pligt, J. (2016). What Factors Moderate Self-Other Discrepancies in Decision Making? Results from a Vaccination Scenario. Spanish Journal of Psychology, 19(e52), 1-11.﻿ https://doi.org/10.1017/sjp.2016.50
24. Pollmann, M. M. H., Potters, J., & Trautmann, S. T. (2014). Risk taking by agents: The role of ex-ante and ex-post accountability. Economics Letters, 123(3), 387–390. <https://doi.org/10.1016/j.econlet.2014.04.004>
25. Polman, E. (2012). Self-other decision making and loss aversion. Organizational Behavior and Human Decision Processes, 119(2), 141–150. <https://doi.org/10.1016/j.obhdp.2012.06.005>
26. Reynolds, D. B., Joseph, J., Sherwood, R. (2009). Risky shift versus cautious shift: determining differences in risk taking between private and public management decision-making. Journal of Business & Economics Research, 7(1), 63-78. https://doi.org/10.19030/jber.v7i1.2251
27. Rigoli, F., Preller, K. H., & Dolan, R. J. (2018). Risk preference and choice stochasticity during decisions for other people. Cognitive, Affective and Behavioral Neuroscience, 18(2), 331–341. <https://doi.org/10.3758/s13415-018-0572-x>
28. Stone, E. R., & Allgaier, L. (2008). A Social Values Analysis of Self-Other Differences in Decision Making Involving Risk. Basic & Applied Social Psychology, 30(2), 114–129. <https://doi.org/10.1080/01973530802208832>
29. Stone, E. R., Choi, Y., de Bruin, W. B., & Mandel, D. R. (2013). I can take the risk, but you should be safe: Self-other differences in situations involving physical safety. Judgment and Decision Making, 8(3), 250–67. Retrieved from <http://journal.sjdm.org/10/10907/jdm10907.html>
30. Sun, Q., Liu, Y., Zhang, H., & Lu, J. (2016). Increased Social Distance Makes People More Risk Neutral. The Journal of Social Psychology, 00(00), 00224545.2016.1242471. <https://doi.org/10.1080/00224545.2016.1242471>
31. Tang, M. Y., Shahab, L., Robb, K. A., & Gardner, B. (2016). Are parents more willing to vaccinate their children than themselves? Journal of Health Psychology, 21(5), 781–787. <https://doi.org/10.1177/1359105314539527>
32. Teger, & Kogan. (1971). The effect of a reciprocal decision rule on decisions for another person. Proceedings of the 79th Annual Convention of the American Psychological Association, 6.
33. Tunney, R. J. (2015). Framing effects in surrogate decision making. Unpublished data.
34. Vlaev, I., Wallace, B., Wright, N., Nicolle, A., Dolan, P., & Dolan, R. (2017). Other People's Money: The Role of Reciprocity and Social Uncertainty in Decisions for Others. Journal of Neuroscience, Psychology, and Economics, 10(2&3), 59-80. ﻿http://dx.doi.org/10.1037/npe0000063
35. Wray, L. D., & Stone, E. R. (2005). The role of self-esteem and anxiety in decision making for self versus others in relationships. Journal of Behavioral Decision Making, 18(2), 125–144. <https://doi.org/10.1002/bdm.490>
36. Zaleska, M., & Kogan, N. (1971). Level of Risk Selected by Individuals and Groups When Deciding for Self and for Others. Sociometry, 34(2), 198–213.
37. Zhang, X., Liu, Y., Chen, X., Shang, X., & Liu, Y. (2017). Decisions for others are less risk-averse in the gain frame and less risk-seeking in the loss frame than decisions for the self. Frontiers in Psychology, 8(SEP), 1–10. <https://doi.org/10.3389/fpsyg.2017.01601>
38. Ziegler, F. V, & Tunney, R. J. (2015). Who’s been framed? Framing effects are reduced in financial gambles made for others. BMC Psychology, 3(1), 9. <https://doi.org/10.1186/s40359-015-0067-2>
39. Zikmund-Fisher, B. J., Sarr, B., Fagerlin, A., & Ubel, P. a. (2006). A matter of perspective: Choosing for others differs from choosing for yourself in making treatment decisions. Journal of General Internal Medicine, 21(6), 618–622. <https://doi.org/10.1111/j.1525-1497.2006.00410.x>
