# Supplementary material for: Do our risk preferences change when we make decisions for others? A meta-analysis of self-other differences in decisions involving risk
Source: PLoS One. 2019 May 8;14(5):e0216566. doi: 10.1371/journal.pone.0216566 (PMC6505775; doi:10.1371/journal.pone.0216566)
Supplement: S2 Appendix — (DOCX) [file pone.0216566.s002.docx]

Supplementary File 2: Characteristics and effect sizes of all studies

| Author | Year | Study | Domain | Frame | Recipient | Design | Outcome | Publication | N (total) | g | Variance |
| --- | --- | --- | --- | --- | --- | --- | --- | --- | --- | --- | --- |
| Andersson et al | 2013 | 1 | Financial | Gain | Group | Between | Real | Unpublished | 437 | 0.082 | 0.095 |
| Andersson et al | 2014 | 1 | Financial | Gain | Stranger | Between | Real | Published | 168.5 | -0.007 | 0.153 |
| Andersson et al | 2014 | 1 | Financial | Gain with Loss | Stranger | Between | Real | Published | 168.5 | 0.190 | 0.153 |
| Batteux et al | 2017a | 1 | Financial | Gain | Stranger | Within | Real | Published | 99 | 0.077 | 0.141 |
| Batteux et al | 2017a | 1 | Financial | Gain | Close | Within | Real | Published | 99 | 0.365 | 0.142 |
| Batteux et al | 2017b | 1 | Financial | Gain with Loss | Stranger | Within | Real | Unpublished | 35 | 0.469 | 0.237 |
| Batteux et al | 2017b | 2 | Financial | Gain with Loss | Stranger | Within | Real | Unpublished | 79 | 0.378 | 0.159 |
| Batteux et al | 2017b | 3 | Medical | Gain | Stranger | Within | Hypothetical | Unpublished | 36 | -0.021 | 0.231 |
| Batteux et al | 2017b | 3 | Medical | Loss with Gain | Stranger | Within | Hypothetical | Unpublished | 36 | -0.328 | 0.232 |
| Batteux et al | 2017b | 4 | Financial | Gain | Stranger | Within | Real | Unpublished | 46 | 0.481 | 0.208 |
| Batteux et al | 2017b | 4 | Financial | Loss | Stranger | Within | Real | Unpublished | 46 | 0.081 | 0.205 |
| Batteux et al | 2019 | 2 | Financial | Gain with Loss | Stranger | Within | Real | Published | 36 | 0.017 | 0.231 |
| Batteux et al | 2019 | 2 | Medical | Loss with Gain | Stranger | Within | Hypothetical | Published | 36 | -0.252 | 0.232 |
| Beisswanger et al | 2003 | 1 | Interpersonal | Gain with Loss | Close | Between | Hypothetical | Published | 134 | 0.344 | 0.172 |
| Beisswanger et al | 2003 | 2 | Interpersonal | Gain with Loss | Close | Between | Hypothetical | Published | 182 | 0.422 | 0.149 |
| Beisswanger et al | 2003 | 3 | Interpersonal | Gain with Loss | Close | Between | Hypothetical | Published | 81 | 0.572 | 0.223 |
| Benjamin & Robbins | 2007 | 1 | Financial | Gain | Close | Within | Hypothetical | Published | 36 | 0.138 | 0.231 |
| Author | Year | Study | Domain | Frame | Recipient | Design | Outcome | Publication | N (total) | g | Variance |
| Benjamin & Robbins | 2007 | 1 | Financial | Loss | Close | Within | Hypothetical | Published | 36 | -0.371 | 0.233 |
| Carroll et al | 2017 | 1 | Medical | Loss with Gain | Close | Between | Hypothetical | Published | 701 | -0.230 | 0.055 |
| Carroll et al | 2017 | 1 | Medical | Loss with Gain | Close | Between | Hypothetical | Published | 701 | -0.033 | 0.064 |
| Carstendsdottir | 2015 | 1 | Financial | Gain | Close | Between | Hypothetical | Unpublished | 37 | 0.119 | 0.315 |
| Colby | 2010 | 2 | Financial | Gain | Close | Between | Hypothetical | Unpublished | 260.5 | 0.201 | 0.124 |
| Dore et al | 2014 | 1 | Medical | Gain | Close | Within | Hypothetical | Published | 100 | -0.406 | 0.142 |
| Dore et al | 2014 | 2 | Medical | Gain | Close | Within | Hypothetical | Published | 216 | -0.322 | 0.097 |
| Eriksen & Kvaloy | 2010 | 1 | Financial | Gain with Loss | Stranger | Between | Real | Published | 320 | -0.155 | 0.111 |
| Eriksen et al | 2017 | 1 | Financial | Gain | Stranger | Within | Real | Unpublished | 190 | -0.057 | 0.102 |
| Eriksen et al | 2017 | 1 | Financial | Gain with Loss | Stranger | Within | Hypothetical | Unpublished | 190 | 0.040 | 0.102 |
| Fleming & Slank | 2015 | 1 | Interpersonal | Gain with Loss | Stranger | Between | Hypothetical | Published | 165 | 0.337 | 0.155 |
| Fullbrunn & Luhan | 2015 | 1 | Financial | Gain with Loss | Group | Within | Real | Unpublished | 175 | -0.279 | 0.107 |
| Fullbrunn & Luhan | 2015 | 2 | Financial | Gain with Loss | Stranger | Within | Real | Unpublished | 34 | -0.015 | 0.237 |
| Garcia-Retamero & Galesic | 2012 | 1 | Medical | Loss with Gain | Close | Within | Hypothetical | Published | 40 | -1.317 | 0.242 |
| Haavik & Zeiler | 2010 | 1 | Financial | Gain | Stranger | Between | Real | Unpublished | 80 | 0.636 | 0.225 |
| Humphrey & Renner | 2011 | 1 | Financial | Gain | Stranger | Between | Real | Unpublished | 87 | -0.164 | 0.212 |
| Humphrey & Renner | 2011 | 1 | Financial | Gain | Close | Between | Real | Unpublished | 99 | -0.050 | 0.198 |
| Lu et al | 2018 | 1 | Financial | Gain | Close | Between | Hypothetical | Published | 132 | -0.252 | 0.175 |
| Lu et al | 2018 | 1 | Financial | Loss | Close | Between | Hypothetical | Published | 132 | 0.090 | 0.174 |
| Lu et al | 2018 | 2 | Financial | Gain | Close | Between | Hypothetical | Published | 119 | -0.404 | 0.185 |
| Author | Year | Study | Domain | Frame | Recipient | Design | Outcome | Publication | N (total) | g | Variance |
| Lu et al | 2018 | 2 | Financial | Loss | Close | Between | Hypothetical | Published | 119 | -0.106 | 0.183 |
| Montinari & Rancan | 2013 | 1 | Financial | Gain with Loss | Stranger | Within | Real | Unpublished | 254 | -0.027 | 0.088 |
| Montinari & Rancan | 2013 | 1 | Financial | Gain with Loss | Close | Within | Real | Unpublished | 254 | -0.342 | 0.089 |
| Oliver | 2013 | 1 | Medical | Loss with Gain | Stranger | Within | Hypothetical | Published | 60 | -0.604 | 0.184 |
| Palmer | 2014 | 1 | Interpersonal | Gain with Loss | Close | Between | Hypothetical | Unpublished | 230 | 0.550 | 0.171 |
| Petrova et al | 2016 | 1 | Medical | Gain | Close | Within | Hypothetical | Published | 114 | -0.262 | 0.118 |
| Pollmann et | 2014 | 1 | Financial | Gain with Loss | Stranger | Between | Real | Published | 222 | 0.256 | 0.173 |
| Polman | 2012 | 3 | Financial | Gain with Loss | Stranger | Between | Real | Published | 140 | 0.388 | 0.169 |
| Reynolds et al | 2009 | 1 | Financial | Gain with Loss | Group | Within | Real | Published | 85 | -0.323 | 0.153 |
| Reynolds et al | 2009 | 2 | Financial | Gain with Loss | Group | Within | Real | Published | 42 | 0.000 | 0.214 |
| Rigoli et al | 2018 | 1 | Financial | Gain | Stranger | Within | Real | Published | 40 | 0.379 | 0.221 |
| Stone & Allgaier | 2008 | 3 | Interpersonal | Gain with Loss | Close | Between | Hypothetical | Published | 55 | 0.155 | 0.275 |
| Stone & Allgaier | 2008 | 3 | Interpersonal | Gain with Loss | Stranger | Between | Hypothetical | Published | 55 | 0.249 | 0.272 |
| Stone et al | 2013 | 1 | Interpersonal | Gain with Loss | Close | Between | Hypothetical | Published | 180 | 0.777 | 0.153 |
| Stone et al | 2013 | 1 | Medical | Gain with Loss | Close | Between | Hypothetical | Published | 180 | -0.671 | 0.152 |
| Sun et al | 2016 | 1 | Financial | Gain | Stranger | Between | Hypothetical | Published | 95 | 1.074 | 0.216 |
| Sun et al | 2016 | 1 | Financial | Loss | Stranger | Between | Hypothetical | Published | 95 | -0.122 | 0.202 |
| Tang et al | 2016 | 1 | Medical | Gain | Close | Within | Hypothetical | Published | 245 | -0.150 | 0.090 |
| Tang et al | 2016 | 1 | Medical | Gain | Close | Within | Hypothetical | Published | 245 | -0.043 | 0.090 |
| Author | Year | Study | Domain | Frame | Recipient | Design | Outcome | Publication | N (total) | g | Variance |
| Teger & Kogan | 1971 | 1 | Financial | Gain with Loss | Stranger | Between | Real | Unpublished | 50 | -0.065 | 0.237 |
| Teger & Kogan | 1971 | 1 | Financial | Gain with Loss | Close | Between | Real | Unpublished | 50 | -0.144 | 0.238 |
| Tunney | 2015 | 1 | Financial | Gain | Stranger | Within | Hypothetical | Unpublished | 59 | 0.204 | 0.182 |
| Tunney | 2015 | 1 | Financial | Gain | Close | Within | Hypothetical | Unpublished | 59 | -0.117 | 0.182 |
| Tunney | 2015 | 1 | Financial | Loss | Stranger | Within | Hypothetical | Unpublished | 59 | 0.099 | 0.182 |
| Tunney | 2015 | 1 | Financial | Loss | Close | Within | Hypothetical | Unpublished | 59 | -0.157 | 0.182 |
| Tunney | 2015 | 2 | Financial | Gain | Stranger | Between | Hypothetical | Unpublished | 90 | 0.050 | 0.207 |
| Tunney | 2015 | 2 | Financial | Loss | Stranger | Between | Hypothetical | Unpublished | 90 | -0.611 | 0.212 |
| Vlaev et al | 2017 | 2 | Financial | Gain | Stranger | Within | Real | Published | 40 | 0.270 | 0.220 |
| Vlaev et al | 2017 | 2 | Financial | Loss | Stranger | Within | Real | Published | 40 | 0.000 | 0.219 |
| Wray & Stone | 2005 | 1 | Interpersonal | Gain with Loss | Close | Within | Hypothetical | Published | 214 | 1.031 | 0.103 |
| Zaleska & Kogan | 1971 | 1 | Financial | Gain with Loss | Group | Within | Real | Published | 54 | -0.252 | 0.265 |
| Zhang et al | 2017 | 1 | Financial | Gain | Stranger | Within | Real | Published | 57 | 0.343 | 0.186 |
| Zhang et al | 2017 | 1 | Financial | Loss | Stranger | Within | Real | Published | 57 | -0.447 | 0.187 |
| Zhang et al | 2017 | 2 | Financial | Gain | Stranger | Within | Real | Published | 82 | 0.383 | 0.156 |
| Zhang et al | 2017 | 2 | Financial | Loss | Stranger | Within | Real | Published | 82 | -0.861 | 0.162 |
| Zhang et al | 2017 | 2 | Financial | Gain | Close | Within | Real | Published | 82 | 0.166 | 0.155 |
| Zhang et al | 2017 | 2 | Financial | Loss | Close | Within | Real | Published | 82 | -0.323 | 0.156 |
| Ziegler & Tunney | 2015 | 1 | Financial | Gain | Stranger | Within | Real | Published | 73 | 0.208 | 0.164 |
| Ziegler & Tunney | 2015 | 1 | Financial | Loss | Stranger | Within | Real | Published | 73 | -0.197 | 0.164 |
| Zikmund-Fisher et al | 2007 | 1 | Medical | Gain with Loss | Stranger | Between | Hypothetical | Published | 1183 | -0.180 | 0.057 |
| Zikmund-Fisher et al | 2007 | 1 | Medical | Gain with Loss | Close | Between | Hypothetical | Published | 1215 | -0.280 | 0.058 |
