# Supplementary material for: Do our risk preferences change when we make decisions for others? A meta-analysis of self-other differences in decisions involving risk
Source: PLoS One. 2019 May 8;14(5):e0216566. doi: 10.1371/journal.pone.0216566 (PMC6505775; doi:10.1371/journal.pone.0216566)
Supplement: S3 Appendix — (DOCX) [file pone.0216566.s003.docx]

Supplementary File 3: Coding frame for methodological and theoretical moderators

| Moderator | Code | Definition |
| --- | --- | --- |
| Theoretical moderator: Domain | Financial (*k*=54) | Decisions where the recipient can win or lose money |
|  | Interpersonal  (*k*=9) | Decisions concerning romantic scenarios between two people: the recipient of the decision and a potential mate or partner |
|  | Medical/Safety (*k*=15) | Decisions concerning the recipient’s health or safety |
| Theoretical moderator: Frame | Gain (*k*=30) | Decisions which place the decision-maker in a gain frame relative to their current position (e.g. winning money or finding a partner) |
|  | Loss (*k*=12) | Decisions which place the decision-maker in a loss frame relative to their current position (e.g. losing money or being ill) |
|  | Gain with Loss (*k*=29) | Decisions which place the decision-maker in a gain frame relative to their current position but which can incur a loss (e.g. investment tasks where the final outcome will be a gain, but losses might be incurred during) |
|  | Gain with Loss (*k*=7) | Decisions which place the decision-maker in a loss frame relative to their current position but which can result in a gain (e.g. taking a treatment to attempt to recover from an illness) |
| Theoretical moderator: Recipient | Stranger (*k*=39) | Recipient who the decision-maker is not familiar with or has not built a relationship with (unknown other, fellow participant, unidentified patient, patient with no evidence of a doctor-patient relationship) |
|  | Close other (*k*=34) | Recipient who the decision-maker has a relationship with (friend, relative, long-term patient) |
|  | Group (*k*=5) | Decision where there are several recipients (more than one person) |
| Methodological moderator: Outcome | Real (*k*=37) | Decisions which had a real outcome: the recipient received the outcome of the choice |
|  | Hypothetical (*k*=41) | Decisions which had a hypothetical outcome: the recipient did not receive the outcome of the choice |
| Methodological moderator: Design | Between-subjects (*k*=34) | Studies where one group of participants made choices for themselves and another group of participants made choices for someone else – self-other differences were elicited between groups |
|  | Within-subjects (*k*=44) | Studies where one group made choices for themselves and choices for someone else – self-other differences were elicited within one group |
| Methodological | Published (*k*=51) | Published journal article |
| moderator: Publication status | Unpublished (*k*=27) | Dissertations/thesis, working paper, conference proceeding, unpublished data |
