# Supplementary material for: Do our risk preferences change when we make decisions for others? A meta-analysis of self-other differences in decisions involving risk
Source: PLoS One. 2019 May 8;14(5):e0216566. doi: 10.1371/journal.pone.0216566 (PMC6505775; doi:10.1371/journal.pone.0216566)
Supplement: S4 Appendix — (DOCX) [file pone.0216566.s004.docx]

Supplementary File 4: Statistical differences between self and other decisions

|  | k | g | CI | Q | p |
| --- | --- | --- | --- | --- | --- |
| **Main Effects** |  |  |  |  |  |
| All studies | 49 | 0.009 | [-0.092, 0.109] | 42.281 | .705 |
| Financial | 31 | 0.036 | [-0.095, 0.167] | 11.433 | .533 |
| Medical | 11 | -0.297 | [-0.481, -0.112] | 7.231 | **.002** |
| Interpersonal | 8 | 0.571 | [0.296, 0.847] | 3.838 | **<.001** |
| **Moderator: Domain** |  |  |  | 27.666 | **<.001** |
| Financial | 54 | 0.010 | [-0.099, 0.120] |  | .852 |
| Medical | 15 | -0.267 | [-0.430, -0.105] |  | **.001** |
| Interpersonal | 9 | 0.554 | [0.285, 0.823] |  | **<.001** |
| **Moderator: Frame** |  |  |  | 13.531 | **.009** |
| Gain | 20 | 0.063 | [-0.078, 0.204] |  | .379 |
| Loss | 12 | -0.264 | [-0.507, -0.022] |  | **.033** |
| Gain with Loss | 29 | 0.101 | [-0.040, 0.241] |  | .159 |
| Loss with Gain | 7 | -0.300 | [-0.535, -0.064] |  | **.013** |
| **Moderator: Recipient** |  |  |  | 1.096 | .778 |
| Stranger | 39 | 0.038 | [-0.092, 0.168] |  | .567 |
| Close other | 34 | -0.047 | [-0.168, 0.073] |  | .440 |
| Group | 5 | -0.076 | [-0.436, 0.283] |  | .677 |
| **Moderator: Outcome** |  |  |  | 0.401 | .818 |
| Real | 37 | 0.016 | [-0.113, 0.146] |  | .803 |
| Hypothetical | 41 | -0.034 | [-0.149, 0.081] |  | .560 |
| **Moderator: Design** |  |  |  | 0.927 | .629 |
| Between-subjects | 34 | 0.032 | [-0.095, 0.159] |  | .619 |
| Within-subjects | 44 | -0.049 | [-0.166, 0.068] |  | .410 |
| **Moderator: Publication status** |  |  |  | 0.222 | .895 |
| Published | 51 | -0.024 | [-0.129, 0.082] |  | .657 |
| Unpublished | 27 | 0.012 | [-0.136, 0.160] |  | .875 |
| **Medical Domain Analyses** |  |  |  |  |  |
| **Moderator: Frame** |  |  |  | 8.391 | **.015** |
| Gain | 6 | -0.203 | [-0.474, 0.068] |  | .141 |
| Loss with Gain | 7 | -0.300 | [-0.535, -0.064] |  | **.013** |
| **Moderator: Recipient** |  |  |  | 10.470 | **.005** |
| Stranger | 4 | -0.319 | [-0.776, 0.138] |  | .171 |
| Close other | 11 | -0.260 | [-0.434, -0.086] |  | **.003** |
| **Moderator: Design** |  |  |  | 10.657 | **.005** |
| Between-subjects | 5 | -0.228 | [-0.454, -0.001] |  | **.049** |
| Within-subjects | 10 | -0.310 | [-0.543, -0.076] |  | **.009** |
| **Financial Domain Analyses** |  |  |  |  |  |
| **Moderator: Frame** |  |  |  | 8.323 | **.040** |
| Gain | 24 | 0.163 | [-0.003, 0.328] |  | .054 |
| Loss | 12 | -0.264 | [-0.507, -0.022] |  | **.033** |
| Gain with Loss | 18 | -0.020 | [-0.202, 0.162] |  | .831 |
| **Moderator: Outcome** |  |  |  | 0.064 | .969 |
| Real | 37 | 0.016 | [-0.113, 0.146] |  | .803 |
| Hypothetical | 17 | -0.005 | [-0.207, 0.198] |  | .965 |
| **Moderator: Recipient** |  |  |  | 1.270 | .736 |
| Stranger | 33 | 0.059 | [-0.080, 0.198] |  | .404 |
| Close other | 16 | -0.065 | [-0.268, 0.137] |  | .527 |
| Group | 5 | -0.076 | [-0.436, 0.436] |  | .678 |
| **Moderator: Design** |  |  |  | 0.263 | .877 |
| Between-subjects | 21 | 0.045 | [-0.134, 0.224] |  | .623 |
| Within-subjects | 33 | -0.010 | [-0.148, 0.128] |  | .885 |
| **Moderator: Publication status** |  |  |  | 0.035 | .983 |
| Published | 30 | 0.011 | [-0.141, 0.164] |  | .884 |
| Unpublished | 24 | 0.009 | [-0.148, 0.166] |  | .907 |
